# A Voting-Based Sequential Pattern Recognition Method

**DOI:** 10.1371/journal.pone.0076980

**Published:** 2013-10-14

**Authors:** Koichi Ogawara, Masahiro Fukutomi, Seiichi Uchida, Yaokai Feng

**Affiliations:** 1 Faculty of Systems Engineering, Wakayama University, Wakayama-shi, Wakayama, Japan; 2 Graduate School of Information Science and Electrical Engineering, Kyushu University, Nishi-ku, Fukuoka, Japan; 3 Faculty of Information Science and Electrical Engineering, Kyushu University, Nishi-ku, Fukuoka, Japan; Cinvestav-Merida, Mexico

## Abstract

We propose a novel method for recognizing sequential patterns such as motion trajectory of biological objects (i.e., cells, organelle, protein molecules, etc.), human behavior motion, and meteorological data. In the proposed method, a local classifier is prepared for every point (or timing or frame) and then the whole pattern is recognized by majority voting of the recognition results of the local classifiers. The voting strategy has a strong benefit that even if an input pattern has a very large deviation from a prototype locally at several points, they do not severely influence the recognition result; they are treated just as several incorrect votes and thus will be neglected successfully through the majority voting. For regularizing the recognition result, we introduce partial-dependency to local classifiers. An important point is that this dependency is introduced to not only local classifiers at neighboring point pairs but also to those at distant point pairs. Although, the dependency makes the problem non-Markovian (i.e., higher-order Markovian), it can still be solved efficiently by using a graph cut algorithm with polynomial-order computations. The experimental results revealed that the proposed method can achieve better recognition accuracy while utilizing the above characteristics of the proposed method.

## Introduction

Recognition of sequential patterns, such as motion trajectory of biological objects (i.e., cells, organelle, protein molecules, etc.), human behavior motion, and meteorological data, is one of active research topics in pattern recognition. Traditional methods evaluate distance (or similarity) between an input pattern and a prototype by, for example, dynamic programming (DP) and Hidden Markov Models (HMM). The class label of the prototype with the minimum distance is determined as the class of the input pattern. It should be noted that, for recognizing a pattern whose length is 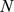, the distance is generally defined as *accumulation* of 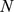 local distances, which are calculated individually at every point 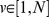. We use the simple term “point” to express the address of each element of various sequential patterns. The point can be interpreted as the frame number for video and the time index for temporal patterns.

**Figure 1:**
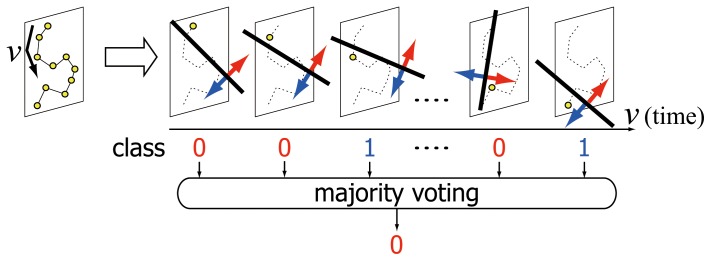
Sequential pattern recognition by majority voting.

In this paper, we propose a novel method for recognizing sequential patterns, where a local classifier is prepared for every point and then the whole pattern is finally recognized based on majority voting of the class labels given by the local classifiers. Figure 1 illustrates this idea. When we consider a two-class recognition task, a two-class local classifier will be prepared at each point. Note that we can use an arbitrary classifier as the local classifier. Here, we will consider mainly a two-class problem, just for simplicity. We can deal with multi-class problems by, for example, combining the two-class classifier like SVM.

We can expect theoretically that the voting-based recognition framework of Figure 1 will have the following merits. First, if a very large deviation from a prototype happens at several points, its bad influence on the final recognition result will be negligible; this is because they are neglected as just a small number of incorrect votes among many correct votes. In contrast, the traditional distance-based methods will suffer from the large deviation. Second, by using different kinds of local classifiers at several points, the proposed method becomes a “heterogeneous” classifier and can create a complex classification boundary. Third, the proposed method can be used for some kind of application of recognizing a pattern without using the whole pattern. For example, we can realize early recognition [1], [2], where the recognition result is determined at the beginning part of the pattern, just by voting the recognition results of the beginning part.

In the experimental result, it is shown that a simple majority voting method, where the votes from the local classifiers are totally independent, is not satisfactory. This is because the local classifier determines its recognition result just by observing a local part of the pattern and thus the result becomes unstable. If we have incorrect results due to the instability at many points, the final recognition result will be also incorrect even after voting.

In the proposed method, a dependency is introduced between several point pairs 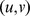 for avoiding the instability. Specifically, we add a penalty when the local recognition results at a certain pair of points 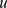 and 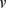 are different. Hereafter, we call the penalty *smoothness term*. The smoothness term can be introduced between not only neighboring point pairs (i.e., 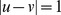) but also some distant point pairs (i.e., 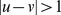). This implies that some training is necessary to determine the point pairs where the smoothness term is to be introduced.

The proposed method is formulated as an optimal label assignment problem to determine the class label at every point 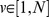. Let 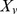 denote the label variable at the point 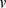 and its value equals to 0 or 1 for two-class recognition. For the simplicity, we call 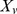 the class label unless confusion arises. The optimized class labels 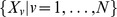 are voted to have the final result. The objective function of the label assignment problem is the sum of all the outputs of the local classifier and the smoothness term. Due to the smoothness term, it is impossible to determine the label class 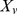 independently. Moreover, the optimization problem becomes non-Markovian due to the smoothness term between distant point pairs and thus becomes intractable by the optimization methods (such as DP) which assume the Markovian property of the problem. Note that we call the popular first-order Markovian property Markovian and the higher-order Markovian property *non-Markovian* throughout this paper.

In this paper, we use a graph cut algorithm [3]. Graph cut can deal with a specific kind of optimization problem very efficiently, and thus has been used for image restoration [4], [5], object recognition [6], shape matching [7], [8], segmentation [8]–[12], etc. The graph cut algorithm can provide the globally optimal solution for our non-Markovian label assignment problem with few computations.

An important point of this paper is that the proposed method is a realization of *classifier ensemble* based on global optimization. When assembling outputs of local classifiers, we can introduce non-Markovian dependency as noted above. Furthermore, this dependency is controlled in a globally optimal manner. On this point, the proposed method is different from simple classifier ensemble methods [13], Boosting, and Bagging methods.

## Methods

In this section, the proposed method is described in a two-step manner; first, a simplified version of the proposed method, called *independent voting*, is introduced. Then, a more sophisticated version, called *dependent voting*, is introduced.

### Independent Voting

The independent voting method is a direct application of simple majority voting to sequential pattern recognition. Let 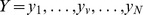 denote an input sequential pattern, where 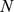 is the length of 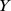 and 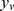 is a 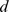-dimensional feature vector.

In the independent voting method, a local classifier 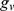 is prepared for each point 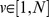 in advance. Then for the input pattern 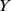, two-class recognition is performed at each 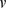 by 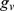 and then resulting 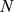 class labels are aggregated by the majority voting for the final recognition result. Without loss of generality, we hereafter assume that the local classifier 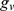 makes its classification by only using the single feature vector 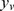. Any classifier, such as simple nearest neighbor classifier and quadratic classifier, can be used for the local classifier.

**Figure 2:**
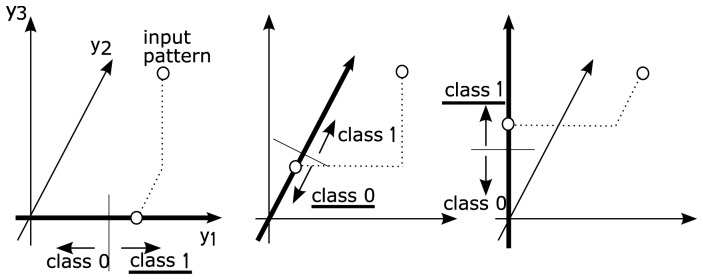
Recognition by the independent voting method. (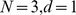).

Figure 2 illustrates the independent voting method for 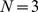 and 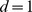. For the input pattern depicted as a “
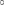
” in this 3-dimensional pattern space, class labels are determined as 1, 0, and 1 at 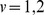, and 3, respectively, using local classifiers whose classification boundary is shown on each axis. The input pattern is finally recognized as class 1 by majority voting.

The assignment of the class label for each point is formulated as a minimization problem as follows. Let 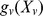 denote the cost of recognizing 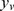 as 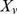 by the local classifier 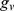. (Precisely speaking, 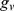 should be denoted as 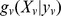, but we prefer the simpler notation 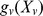.) The optimal label assignment problem is then considered as a minimization problem of the criterion 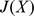 for the label assignment 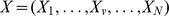


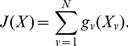
(1)


Clearly, this minimization problem can be easily solved independently at each 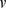, since there is no mutual dependency between two labels 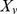 and 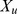 (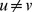).

The problem of the independent voting method is its instability. Since the local classifier 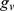 relies on the local feature 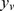 (i.e., a local part of 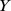), incorrect class labels might be assigned to some points. This instability becomes very crucial, especially when the difference between two classes is very slight and the number of incorrect votes tends to be close to the number of correct votes.

### Dependent Voting

In the dependent voting method, partial dependency is newly introduced between some point pairs, for stabilizing (i.e., regularizing) the label assignment. This dependency is represented as a kind of penalty, called the smoothness term, for forcing 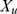 and 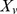 to have the same class labels. Roughly speaking, the smoothness term can eliminate short misrecognized segments. The effect of the smoothness term will be detailed later.

With the smoothness term, the criterion function (1) is extended as follows:
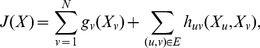
(2)where 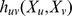 represents the smoothness term between 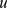 and 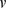 and 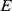 represents a set of point pairs having the smoothness term.

The smoothness term is designed to be larger for 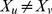 and smaller for 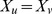. The following equation is an example of the smoothness term:
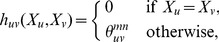
(3)where 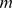 and 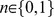 are the class labels assigned to the variables 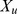 and 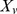, respectively, and 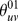 and 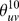 are non-negative constants. Clearly, if 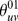 becomes larger, it is more difficult for 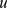 and 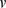 to have the labels 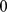 and 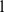, respectively. The value of 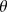 can be determined automatically by some training algorithm (see Section “Training Smoothness Term”) or manually.

We can introduce the smoothness term to not only neighboring point pairs (i.e., 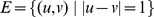) but also distant point pairs (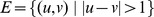). The smoothness term for the distant point pairs is useful, for example, when the mutual dependency between the start point and the end point should be considered. Most traditional sequential pattern recognition methods, such as DP and HMM, cannot deal with such a distant dependency because the distant dependency violates the Markovian property of the problem. In the proposed method, graph cut is used instead of those traditional tools for dealing with the distant dependency, as detailed in Section “Globally Optimal Assignment Using Graph Cut”.

### Effect of Smoothness Terms

**Figure 3:**
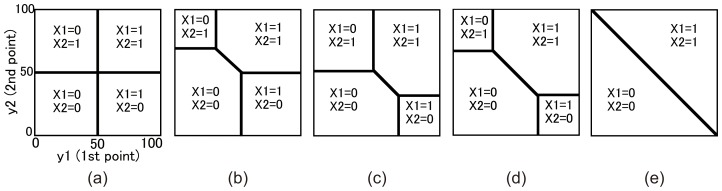
Example of classification boundary for 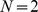. (a) Independent voting (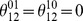), (b) 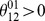 and 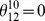, (c) 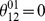 and 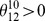, (d) 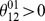 and 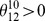, (e) Fully-dependent voting (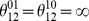).

Figure 3 shows the classification boundary for 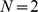 and 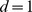. As the local classifier, we assume a simple nearest neighbor classifier whose prototype of class 0 is located at 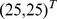 and that of class 1 is 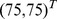. The cost 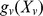 is defined by Euclidean distance as
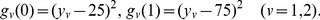
(4)


Figure 3 (a) shows the classification boundary by the independent voting, which is equivalent to the dependent voting with 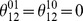. Figures 3 (b)–(e) show the boundaries by the dependent voting with different smoothness terms. These figures show that the larger the smoothness terms are, the smaller the region where the different class labels are assigned, and the larger the region in which the same class labels are assigned. Figure 3 (e) shows the dependent voting with 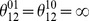. Hereafter, this extreme case is called *fully-dependent voting*. Since no different label is allowed by the infinite smoothness term, all the class labels become the same. In addition, since it is equivalent to the traditional Euclidean distance-based method, that is, the nearest neighbor method, its classification boundary becomes a diagonal line.

**Figure 4:**
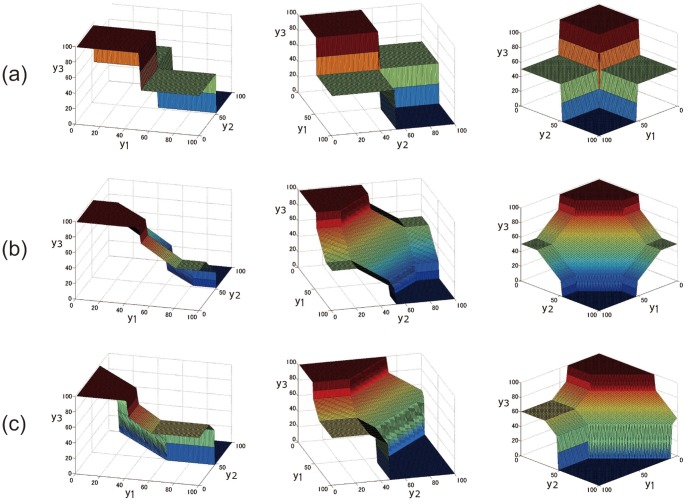
Example of classification boundary for 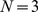 and 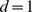 from three different view points. Different from Figure 3, the majority voting was already taken here. (a) Independent voting. (b) Dependent voting with 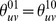. (c) Dependent voting with 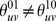.

Figure 4 shows the classification boundary of 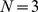, where the prototype of class 0 is located at 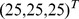 and that of class 1 is 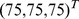 and the recognition cost 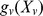 is equal to that of 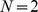. Figures 4 (a), (b), and (c) show the classification boundaries by the independent voting, the dependent voting with a constant smoothness term, and the dependent voting with arbitrary smoothness terms, respectively. Like Figure 3, the smoothness term can control the shape of the classification boundary and a larger smoothness term results in a smoother boundary. Note that, in Figure 4 (b) and (c), positive smoothness terms are introduced not only to neighboring point pairs, but also to the distant point pairs, i.e., 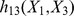 and 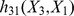. Also note that, although these figures illustrate very low-dimensional cases, the same smoothing effect will appear in a low-dimensional subspace of a higher-dimensional feature space of more realistic sequential patterns.

### Globally Optimal Assignment Using Graph Cut

Since a class label to be assigned to each point is binary, the total number of possible label assignment 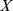 is 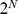. Thus, for a larger 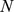, it is impossible to find the globally optimal assignment by the naive exhaustive search. In addition, as already mentioned, it is also impossible to use DP because our problem does not hold Markovian property due to the smoothness terms for distant point pairs.

For our optimization problem, graph cut [3] will be one of the best choices. This is because the globally optimal solution of the criterion function (2) can be obtained with polynomial-order computations, even under the existence of the smoothness terms for distant point pairs. More precisely, if the smoothness terms satisfy the condition called sub-modularity,
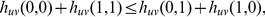
(5)the globally optimal solution can be obtained and it is easily proved that the smoothness term of (3) satisfies this condition.

### Training Smoothness Term

The remaining problem is how to design the smoothness terms. As emphasized above, the parameter value is very crucial factors for determining the classification boundary. Hopefully, those values are to be set up automatically for each application. Although several methods have been proposed for training the smoothness terms in some graph cut problems [4]–[6], no general method has been established yet. In this section, we propose a simple algorithm for training the smoothness terms. Hereafter, we assume a symmetric smoothness term, that is, 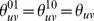.

**Figure 5:**
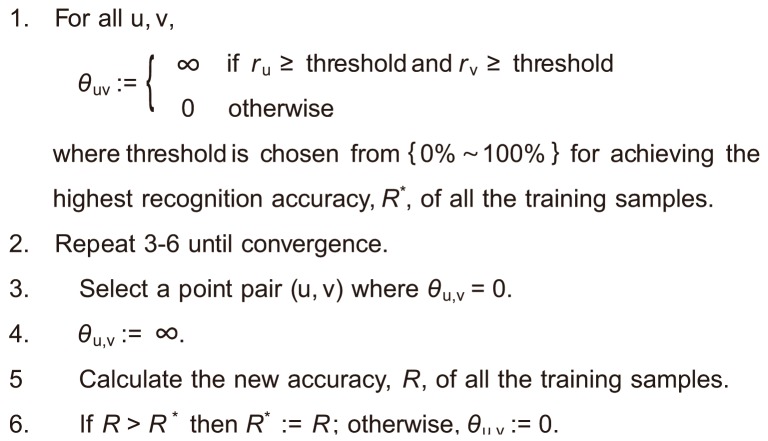
Algorithm for training smoothness terms.

Figure 5 shows the algorithm of training the smoothness terms. This algorithm is similar to an iterative error-correcting strategy for classical perceptrons. First, initial smoothness terms are determined by assigning the infinite smoothness term between points 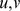 whose local classifiers have recognition accuracies 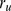 and 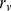 being higher than a threshold. The best threshold achieving the highest recognition accuracy on the training samples is chosen among {0%∼100%}. Note that if the threshold is 0%, the initial smoothness term is reduced to that of the fully-dependent method. Similarly, if the threshold is 100%, the initial smoothness term is reduced to that of the independent method.

Once the initial smoothness terms are determined, they are further optimized iteratively. In the optimization step, the zero smoothness term of a certain point pair is tentatively changed to the infinite smoothness term. Then, for evaluating this change, the recognition accuracy of the training samples is measured. If the accuracy is improved by the change, this change is accepted. Otherwise the change is canceled. This procedure is repeated at point pairs with zero smoothness term until no point pair improves the recognition accuracy.

## Results and Discussion

### Experimental Setup

The proposed method was applied to a two-class online character recognition task for observing its performance, qualitatively and quantitatively because public datasets for rigorous evaluation are available. In online character recognition, each character pattern is represented as a temporal sequence of pen-tip position. That is, it is a kind of sequential pattern recognition and totally different from image-based character recognition (so-called OCR). In other words, the results obtained by the following online character recognition experiment will provide a good reference to any sequential pattern recognition, such as biological motion understanding, by the proposed voting-based method. Ethem Alpaydin Digit dataset (ftp://ftp.ics.uci.edu/pub/machine-learning-databases/pendigits/) that contains about 10,000 online isolated handwritten digit (“0”–“9”) samples was used in the experiment. For each of the 10 classes, about 700 patterns are used as training samples, and the remaining 300 patterns are used as test samples. As preprocessing, linear size normalization and resampling were applied. Each point was simply represented as an 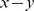 coordinate feature vector, i.e., 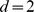. The number of points 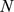 was fixed at 49 by resampling. (An odd number was better to avoid tie break of majority voting.) The local classifier was based on Mahalanobis distance, whose parameters were trained by using the training samples. In other words, the prototype of each class was represented statistically as a sequence of 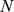 Gaussian distributions. The average recognition rate was evaluated for all 45 two-class tasks of 10 digit classes.

**Figure 6:**
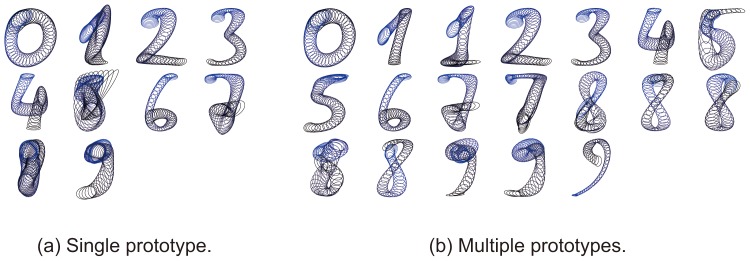
Prototypes used in the experiment. Each ellipsoid illustrates the covariance for Mahalanobis distance at each point.

In the later experiment, multiple prototypes were mainly used. The reason is shown in Figure 6. In the case of single prototype (a), it is observed that too large covariances were often estimated for several digits (e.g., “5” and “8”). This is simply because those digits have variations in writing order and direction. In contrast, in the case of multiple prototypes (b), covariances were estimated reasonably. The multiple prototypes were selected by k-means. After 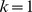 for all classes, k-means was performed at individual classes. Then setting 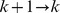 for the class with the largest clustering cost (i.e., the largest quantization error), k-means was performed again. This iteration stops if the clustering cost becomes lower than a fixed threshold at all the classes.

In the multiple prototype scenario, pre-selection of the closest prototype is performed by Mahalanobis distance. This distance is the same as the distance given by the “fully-dependent voting method”, which will be introduced later. For example, for two-class recognition of “5”–“8”, the closest prototype of “5” is selected among 2 prototypes of “5” for each test sample and also that of “8” among 5 prototypes of “8”. Note that a two-class classifier was prepared for all the pairs of prototypes from different classes. Consequently, the total number of the classifiers are more than 45 even for 45 two-class tasks.

The computation time for recognizing all test samples at all 45 two-class tasks (i.e., for 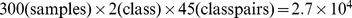 label assignments) was less than 68 seconds by using a notebook computer (CPU Intel Core i7-2960XM 2.70 GHz, Memory 16GB). Therefore, the computation time of the proposed method was about 2.5 ms. This speed is very promising because our optimal label assignment problem is non-Markovian and thus linear time algorithms such as DP optimization are not applicable.

### Quantitative Evaluation

Table 1 shows the average recognition rate of the 45 two-class tasks under different conditions on the smoothness term. The fully-dependent voting method, which was introduced in Section “Effect of Smoothness Terms,” is completely identical to the conventional distance-based recognition method where the discrimination is done by the comparison between 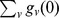 and 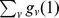. In fact, if 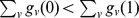, all the labels 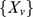 become 0 and then the class 0 wins. In the case of “neighbor-dependent”, the constant smoothness term 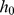 was assigned to neighboring point pairs and zero smoothness term was assigned to the others. The case “optimally-dependent” is our main case where the smoothness terms between not only neighboring pairs but also distant point pairs were trained by the algorithm in Figure 5.

**Table 1:** Average recognition rates (%) for 45 two-class tasks.

prototype	independent	neighbor-independent	fully-dependent*	optimally-dependent
multiple	96.63	97.89	98.12	**98.53**
single	92.67	94.72	94.38	**95.95**

* Equivalent to the conventional distance-based method.

The result shows the superiority of the optimally-dependent voting method over the other methods. First, the optimally-dependent voting (98.53%) was much better than the independent voting (96.63%). This indicates the importance of the smoothness term for voting-based sequential pattern recognition. Although the smoothness term is important, it should be trained appropriately. This fact is indicated by comparing the distance-based method (“fully-dependent”) to voting-based methods; among the three voting-based methods, only the optimally-dependent voting method (98.53%) was better than the fully-dependent voting method (98.12%).

It should be emphasized that the difference between “optimally-dependent” and “fully-dependent”, (i.e., 98.53% and 98.12%) is not trivial. Specifically, by using the optimally-dependent voting method, we could reduce misrecognitions reduced to 78%( = 1.47/1.88). As shown in Table 1, this improvement can also be observed in the single prototype scenario, whose performance was much poorer than the multiple prototype scenario.

**Figure 7:**
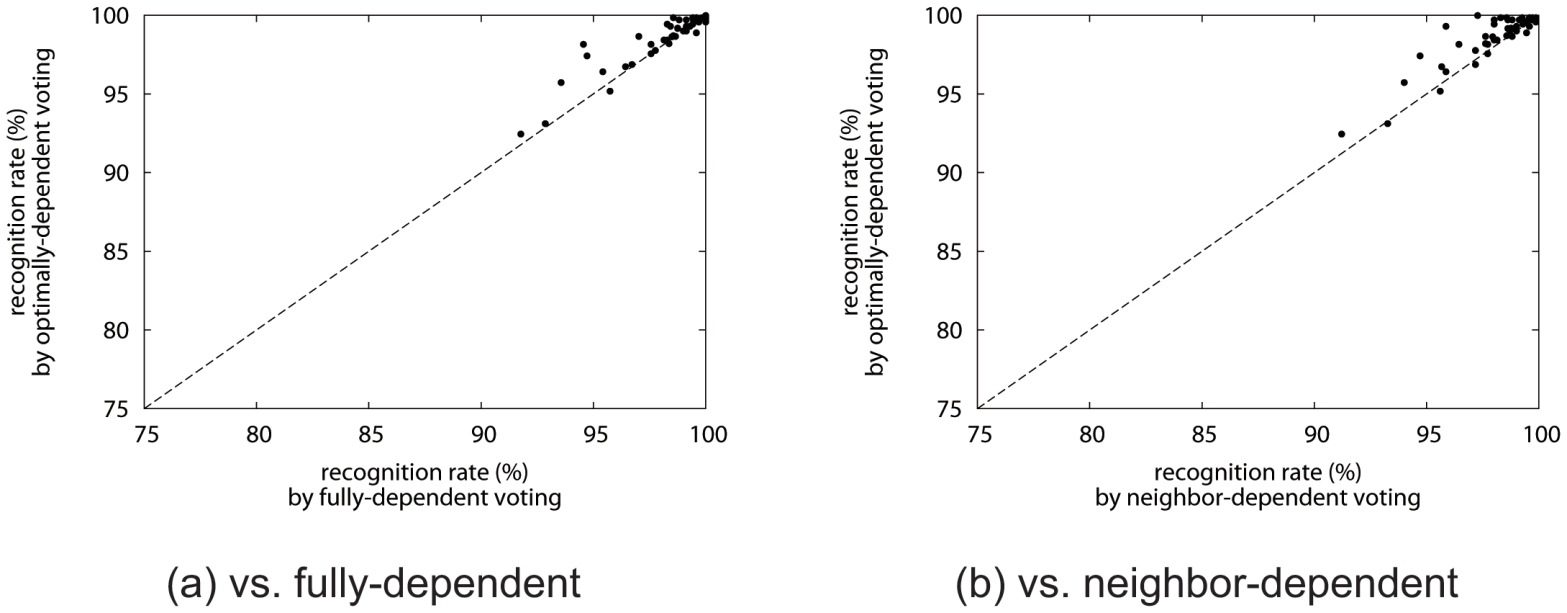
Recognition results of 45 two-class tasks by the optimally-dependent v.s. fully-dependent and neighbor-dependent voting methods.

Figure 7 (a) compares the optimally-dependent voting method with the fully-dependent voting method. The figure proves that the optimally-dependent voting method outperformed in most two-class tasks. Specifically, among 45 two-class tasks, 25 tasks got better, 11 stayed the same, and 8 got worse. The class pairs with the most improved and degraded samples were “7”–“9” (improvement at 25 samples) and “0”–“7” (degradation at 5 samples). It is interesting to note that the top improved pairs are similar-shaped pairs, such as “7”–“9” (25 samples), “0”–“6” (19), “1”–“9” (15), “3”–“9” (11) and “2”–“3” (9). On the other hands, major degradations caused at “0”–“7” (5), “1”–“7” (4) and “2”–“5” (3). The reason of those improvement and degradation will be examined later. Figure 7 (b) compares the optimally-dependent voting method with the neighbor-dependent voting method and shows a similar trend over the 45 two-class tasks.

To evaluate the generalization ability of the proposed method against a small amount of training samples, the same experiment in the multiple prototype scenario was performed by exchanging the training and test samples, i.e., 300 samples for training and 700 samples for test. The average recognition rate of the 45 two-class tasks for the optimally-dependent voting method was slightly degraded from 98.53% to 97.56%, however it still achieved the best recognition rate among the four voting methods.

### Qualitative evaluation

It is interesting to observe how the smoothness terms are trained automatically in the optimally-dependent method. The observation revealed the fact that non-zero smoothness terms do not exist randomly but exist around the parts where shapes of two character patterns are very different and not confusing. This will be because most of local classifiers 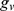 on a non-confusing part will provide correct recognition results and thus the smoothness terms around the part are effective to eliminate the remaining minor incorrect results. In contrast, local classifiers of a confusing part will not show a strong majority of a certain class; for example, about half of local classifiers 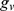 may provide incorrect label. If the smoothness terms are introduced to this confusing part, they have a risk that the correct labels are replaced by the incorrect label.

**Figure 8:**
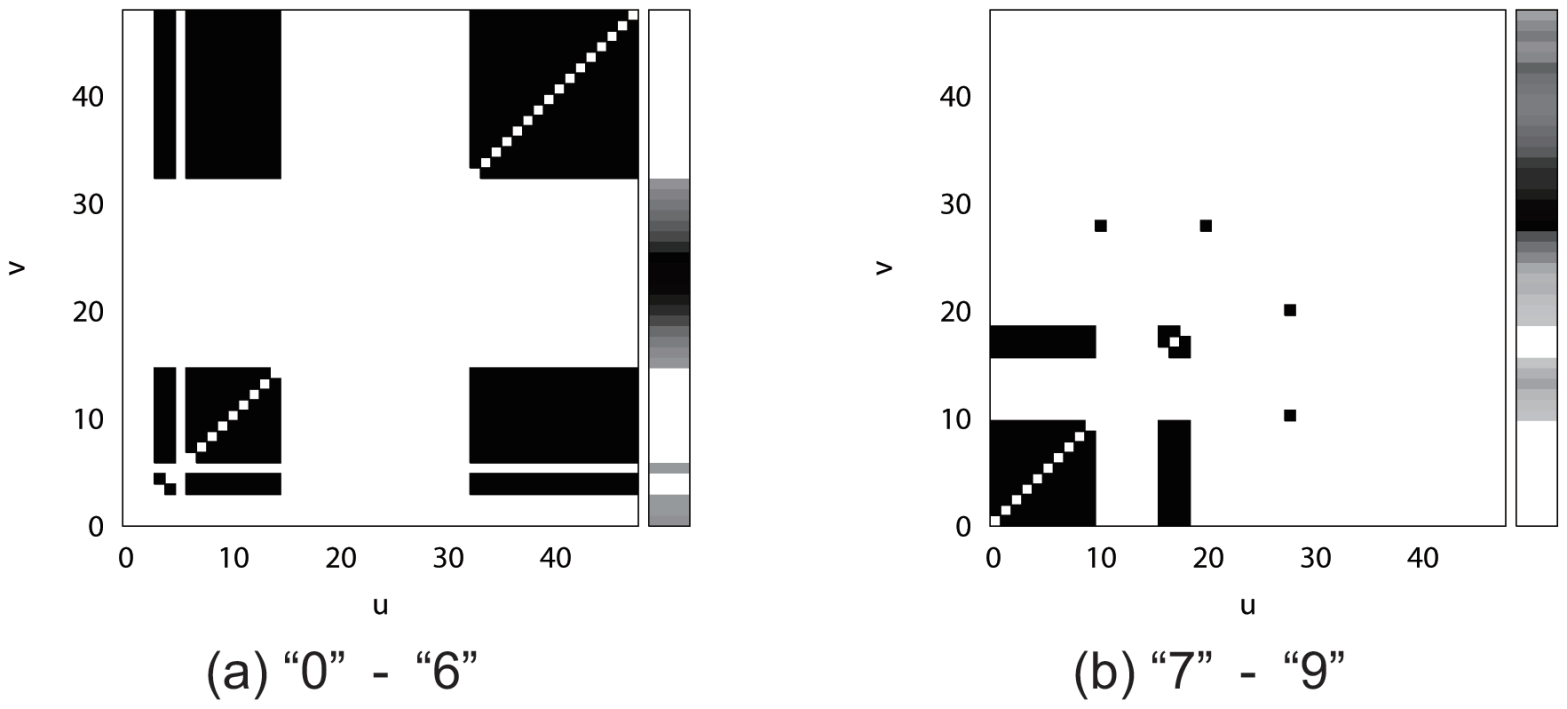
Training results of smoothness terms.

Figure 8 (a) visualizes the smoothness terms trained for “0”–“6” by the optimally-dependent method. In this figure, a dot at 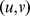 indicates that the point pair 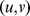 has a non-zero smoothness term. There are many non-zero smoothness terms around the beginning part and the ending part. This can be understood from the fact that the shapes of “0” and “6” are very different in their beginning and ending parts and those parts are better to be forced to have the same label by the smoothness term. In contrast, their middle parts are confusing and thus have a risk that the local classifiers 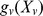 around the part provide many misrecognitions. Since there is no smoothness term around this confusing part, the number of incorrect labels will not increase.

Figure 8 (b) visualizes the smoothness terms trained for “7”–“9” by the optimally-dependent method. Their very beginning parts have the non-zero smoothness terms because the parts are dissimilar. The large remaining parts (vertical stroke parts) have no smoothness term because those parts are confusing. Consequently, the smoothness terms for “7”–“9” are rather sparse than “0”–“6”.

**Figure 9:**
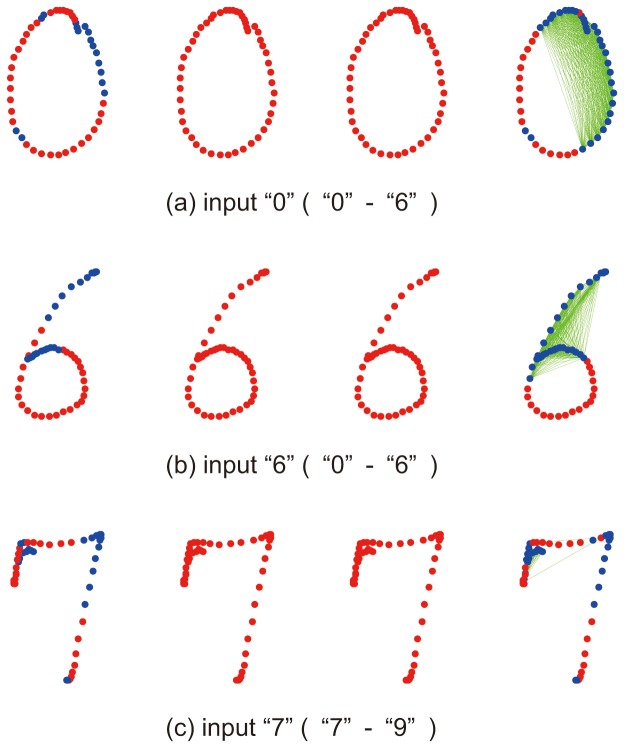
The effect of the distant smoothness terms. From left to right, results by independent voting, neighbor-dependent voting, fully-dependent voting, and optimally-dependent voting methods.

This tendency can be observed from several examples at the rightmost patterns of Figure 9. In this figure, the points with correct labels are depicted by blue circles and those with incorrect labels are by red circles. A point pair with a non-zero smoothness term is connected by a link (green line). The first example, Figure 9 (a), shows the case of recognizing a sample of “0” in “0”–“6” classification task. As shown in Figure 8 (a), there are many non-zero smoothness terms around the beginning and ending parts and no term around the middle part. Without the smoothness term (i.e., the result of the independent method), its confusing middle part is totally misrecognized as “6”. Since this middle part is large, the fully-dependent method results in misrecognition. In contrast, the optimally-dependent method successfully utilizes the correct local recognitions around the beginning and the ending part. The correct recognitions in those parts were locally propagated by the trained smoothness terms, and finally they became the majority. Figure 9 (b) shows the case of recognizing a sample of “6” in the same “0”–“6” classification task. Like Figure 9 (a), the smoothness terms worked for the correct recognition.

Figure 9 (c) shows a result of recognizing a sample of “7” in “7”–“9” classification task. As we observed in Figure 8 (b), this case has a set of non-zero smoothness terms at the beginning part. These smoothness terms forced the local classifiers at the beginning part to have the same recognition result and the correct local classifiers became the majority. Meanwhile, other methods such as the independent voting method, neighbor-dependent voting method and the fully dependent voting method failed in this case.

## Conclusions

In this paper, we have introduced the majority voting strategy to sequential pattern recognition and applied it to online character recognition. Specifically speaking, a two-class classification is done at each point 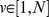 by using a local classifier and the recognition result of the entire pattern is determined by using majority voting of results of local classifiers at all points. This can be formulated as an optimal class label assignment problem where the class labels at all points, 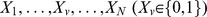, are considered as control variables and the sum of classification costs by local classifiers is considered as a criterion function to be minimized.

We also introduced partial dependency to the local classifiers. If this partial dependency is introduced to a pair of classifiers at 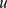 and 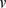, a penalty, called smoothness term, is imposed if those local classifiers have different class labels 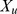 and 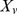. Consequently, the label 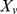 cannot be determined at each 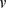 independently; the problem becomes a constrained optimization problem. Moreover, since the smoothness term is introduced to not only neighboring point pairs but also distant point pairs, the traditional DP optimization, which is often used for sequential pattern recognition problems with the Markovian property, cannot be used in our problem. Thus, we have employed graph cut as a more suitable optimization method; using graph cut, we can have the globally optimal solution with polynomial-order computation.

We have the following future work. First, we consider another and more sophisticated method for training the smoothness terms. For example, the steepest decent method may be effective for updating the smoothness terms in a direction of decreasing misrecognition. Second, we extend the proposed method to deal with multi-class problems. Instead of combining two-class classifiers (e.g., 1-vs-others approach), it can be realized by using recent multi-label graph cut methods [14]–[16]. Third, we must deal with nonlinear time warping because it is a very common distortion in sequential patterns. Traditionally, the warping problem is solved by DP; however, we can no longer use DP under the smoothness terms between distant point pairs. Fortunately, in [7], it has been shown that graph cut is promising for the warping problem. Thus it is worth trying to combine the label assignment problem and the time warping problem, and then solve it by graph cut. Fourth, the method should be evaluated on a larger-class problem, which will reveal more non-Markovian dependencies between various pairs of characters with different shapes.
